# Clinical diagnostic performance evaluation of five immunoassays for antibodies to SARS-CoV-2 diagnosis in a real-life routine care setting

**DOI:** 10.11604/pamj.2021.39.3.26471

**Published:** 2021-05-03

**Authors:** Adamu Ishaku Akyala, Jaggu Ruth Awayimbo, Anowai Clementina Ogo, Ndubuisi John Chima, Olusoji Mathew Adeyemi Billyrose, Alaba Ovye Godiya Engom

**Affiliations:** 1 Department of Microbiology, Faculty of Natural and Applied Sciences, Nasarawa State University, Keffi, Nasarawa State, Nigeria,; 2 Primary Health Care Board, Federal Capital Territory Administration, Abuja, Nigeria,; 3 Department of Medical Laboratory Science, Federal University of Lafia, Nasarawa, Nigeria,; 4 Medical Laboratory Services Department, Clinical Chemistry Unit, University of Abuja Teaching Hospital, Gwagwalada, Nigeria,; 5 Department of Histopathology, Medical Laboratory Services, University of Abuja Teaching Hospital, Gwagwalada, Nigeria

**Keywords:** Coronavirus, diagnosis, rapid diagnostic kits, sensitivity, specificity

## Abstract

While molecular techniques remain the gold standard for diagnosis of acute SARS-CoV-2 infection, serological tests have the unique potential to ascertain how much of the population has been exposed to the COVID-19 pathogen. There have been limited published studies to date documenting the performance of SARS-CoV-2 antibody assays in Nigeria and so we evaluated the diagnostic performance of five (5) immunoassay on a set of clinical samples. Five automated immunoassays (2019-nCoV IgG/IgM antibody determination kit, Tigsun COVID-19 combo IgM/IgG rapid test, rapid response COVID-19 IgG/IgM test, COVID-19 IgM-IgG combined antibody rapid test, iChroma COVID-19 Ab) were tested. Three hundred and fourteen specimens were analyzed from health care workers who tested positive PCR for SARS-CoV-2 with symptoms consistent with SARS-CoV-2 receiving treatment at two treatment centres in Nasarawa State from March to September, 2020 with control of 134 health care workers who tested negative PCR for SARS-CoV-2 with no symptoms to SARS-CoV-2. The median patients' age was 40 years (IQR 39.8-41), majority were male and were on admission. The SARS-CoV-2 IgG/IgM antibody evaluated kits had a sensitivity of 33% (2019-nCoV IgG/IgM antibody determination kit), 22% (Tigsun COVID-19 combo IgM/IgG rapid test), 43% (rapid response COVID-19 IgG/IgM test), 44% (COVID-19 IgM-IgG combined antibody rapid test), 25% (iChroma COVID-19 Ab), 100% sensitivity, accuracy of 68.5% and Kappa coefficient of 0.7 and rapid response COVID-19 IgG/IgM test cassette had a sensitivity of 33%, specificity of 100% and accuracy of 72.5% with Kappa coefficient 0.7. The Tigsun COVID-19 combo IgM/IgG rapid test (lateral flow), positive, COVID-19 IgM-IgG combined antibody rapid test and iChroma COVID-19 Ab RT all had sensitivity of zero percent. Serology was complementary to RT-PCR for the diagnosis of COVID-19 at least 14 days after onset of symptoms. The assay panel needs to be improved to serve as an option for the diagnosis of SARS-CoV-2 in resource constrained settings where there are limited molecular diagnostics testing panels.

## Introduction

There are several serological tests available for the diagnosis of Severe Acute Respiratory Syndrome Coronavirus 2 (SARS-CoV-2). Laboratory diagnosis based on reverse transcription-polymerase chain reaction (RT-PCR) remain the gold standard for the rapid diagnosis of acute SARS-CoV-2 infections which is essential for contact tracing and patient management [1]. Several commercial and laboratory developed assays are available but few manufacture-independent evaluations and few comparisons between assays have been published till date [2]. Moreover, the comparison between assays is hampered by the absence of accepted gold standard test as well as our incomplete knowledge of the natural history of SARS-CoV-2 infection [3]. Studies evaluating the concordance between assays are thus needed at this point in the pandemic [4].

Due to the unprecedented pandemic, there has been a quest for an antibody detection testing panel that can detect the virus in blood specimen requiring a sufficient viral load [5]. Over 40 novel SARS-CoV-2-specific antibody testing kits has been developed but there is paucity of information regarding their sensitivity, specificity and kappa level of agreement with the RT-PCR which is the gold standard [6]. There have been huge gaps in the capacity to perform a timely diagnosis using a RT-PCR testing panel and the number of samples in a limited resource setting thus an alternative testing panel as containment of public health strategies especially rapid diagnostic tests (RDTs) which is cost effective, easy to use and adapt to climatic weather and can serve as field base community-based testing panel or point-of-care testing (POCT) is required [7]. This study evaluates the diagnostic performance of five novel antibody-based RDTs for the detection of SARS-CoV-2 in serum and plasma specimens from 134HCW who are positive by RT-PCR to SARS-CoV-2. The sensitivity and specificity of this RDT is compared with RT-PCR as the gold standard.

## Methods

**Study design:** in our study, we evaluated five lateral flow immunoassays for the detection of SARS-CoV-2 antibodies. Patient serum samples used in this study were submitted to the routine Molecular Laboratory of Nasarawa State Infectious Disease and Research Center (NASIRDAC) in for diagnostic evaluation purposes.

**Study period and serum samples:** control serum samples (n=134) included archived anonymous serum obtained from healthy blood donors with no history of SARS-CoV-2 infection, between March 1^st^ and September 2020 (group 1, healthy control). These serum samples were donated to the Nasarawa State Infectious Disease and Research Center (NASIRDAC) in for diagnostic evaluation purposes. Case serum samples were obtained from patients with SARS-CoV-2 infection (n=134) between March 1^st^ and September 2020 (group 2, patients with RT-PCR-positive and group 3, patients with RT-PCR-negative, “clinically diagnosed”, that means patients with pneumonia, showing clinical and radiographic evidence compatible with COVID-19 according to the 5^th^ edition of guideline on diagnosis and treatment of the novel coronavirus pneumonia).

**Real-time PCR assay:** we used three types of automatic extractors to obtain viral RNA from clinical samples, i.e. MagCore HF16 (RBC bioscience, Taipei, Taiwan), Nimbus MicrolabSeegene (Hamilton Company, Bonaduz, Switzerland) and m2000 system (Abbott Molecular Inc. Des Plaines, IL). RNA amplification was made using two real-time PCR platforms, i.e. qCOVID-19 (Genomica, Madrid, Spain) and Allplex 2019-nCoV assay (Seegene, Seoul, South Korea) and we used the CFX96™ (Bio-Rad) real-time detection system. PCR did not have a human extraction control gene target. The extraction control gen target was a phage. These kits were used according to the manufacturer´s instructions for both the handling and the interpretation of the results.

**Rapid diagnostic test:** SARS-CoV-2 antibody test (lateral flow method) is an immunochromatographic assay used for rapid qualitative detection of IgM/IgG in human whole blood serum or plasma samples against SARS-CoV-2 infection. This is a medical diagnostic test that is easy to perform for preliminary or emergency medical screening of SARS-CoV-2 within 20 minutes. The test was performed according to leaflets-protocol provided from the manufacturer in the test kit packet.

**Data analysis:** statistical analysis was carried out using the statistical package STATA/IC version 13.1 (StataCorp, TX, USA). Continuous data are expressed as median and IQR, while categorical data were expressed as frequencies and percentages. Comparisons between variables were made using two-tailed Fisher´s exact test or t test. For these comparisons, a p value less than or equal to 0.05 was considered significant. Sensitivity and specificity were calculated by means of Excel (version 16.0, Microsoft, Washington, USA) using the following definitions in Excel: sensitivity = 100 x [true positive/ (true positive + false negative)] specificity = 100 x [true negative/ (true negative + false positive)]. The agreement between the different serological diagnostic techniques was expressed by the Kappa index and percentage of agreement. A Kappa value of more than 0.75 indicates good agreement between tests, while a value of less than 0.4 indicates poor agreement.

**Ethical consideration:** ethical approval was obtained from the Ethical Review Board of Nasarawa State Ministry of Health. Prior to enrollment in the study, all participants were informed as consent on the objectives and background of the study. Information was provided toward the risks and benefits of the current study. Similarly, a designated questionnaire and data were collected after obtaining returned informed consent. Anonymity and confidentiality of the study participants were maintained.

## Results

There were five testing kits considered for evaluation. Information about the manufacturer, assay commercial name, country of manufacturer, target, volume required, test waiting time, interpretation method and regulatory status of the kits were collected (Table 1). The 2019-nCoV IgG/IgM antibody determination kit, the Tigsun COVID-19 combo IgM/IgG rapid test (lateral flow), rapid response COVID-19 IgG/IgM test cassette (whole blood/serum/plasma), COVID-19 IgM-IgG combined antibody rapid test and iChroma COVID-19 Abtest kits were validated.

**Table 1:** characteristics of five commercially available SARS-CoV-2 antibodies (Ab)-based-detection tests in Nigeria (n=134)

Characteristic	(Ab)- RDTs-1	(Ab)- RDTs-2	(Ab)- RDTs-3	(Ab)- RDTs-4	(Ab)- RDTs-1
Manufacturer	Beijing Diagreat Biotechnologies Co., Ltd	Beijing Tigsun Diagnostics Co., Ltd	BTNX, Inc.	BioMedomics, Inc.	Boditech Inc.
Assay commercial name	2019-nCoV IgG/IgM antibody determination kit	Tigsun COVID-19 combo IgM/IgG rapid test (lateral flow)	Rapid response COVID-19 IgG/IgM test cassette (whole blood/serum/plasma)	COVID-19 IgM-IgG combined antibody rapid test	iChroma COVID-19 Ab
Country of manufacturer	China	China	China	USA	Rep. of Korea
Target	IgM/IgG	IgM/IgG	IgM/IgG	IgM/IgG	IgM/IgG
Volume required	3-4 drops (~90-150 μL)	3-drops (~100-150 μL)	3-drops (~100-150 μL)	2-drops (~100-150 μL)	1-drops (~50-100 μL)
Test waiting time	10-20 min	20min	5-20 min	10 min	5 min
Interpretation	Visual	Visual	Visual	Visual	Visual
Regulatory status (certification)	CE-IVD	CE-IVD; India	CE-IVD	India; CE-IVD	Brazil; CE-IVD

All the kits had certified regulatory status, had visual method of interpretation and all used blood sampling targeting immunoglobulins G and M. The kits had a turnaround time (TAT) of a range between 5-20 minutes though the iChroma COVID-19 Ab test kit had the shortest TAT of five minutes and required only a drop of blood (Table 2). The 2019-nCoV IgG/IgM antibody determination kit had a sensitivity of 33%, 100% specificity, accuracy of 68.5% and Kappa coefficient of 0.7 and rapid response COVID-19 IgG/IgM test cassette had a sensitivity of 33%, specificity of 100% and accuracy of 72.5% with Kappa coefficient 0.7. The Tigsun COVID-19 combo IgM/IgG rapid test (lateral flow), positive, COVID-19 IgM-IgG combined antibody rapid test and iChroma COVID-19 AbRT all had sensitivity of zero percent (Table 2).

**Table 2:** performance evaluation of five SARS-CoV-2 antibodies (Ab)-based-detection tests compared to RT-PCR in Nasarawa State, Nigeria

Antibody detection test		RT-PCR n (134)x		Sensitivity		Specificity		Accuracy	Kappa coefficient
Assay	Result	Positive	Negative	%	95%CI	%	95%CI	%	
2019-nCoV IgG/IgM antibody determination kit	Positive	34	0	33	59.6-98.2	100	89.0-100	62.8	0.7
	Negative	70	30						
Tigsun COVID-19 combo IgM/IgG rapid test (lateral flow)	Positive	20	44	22	29.6-48.2	0	50.0-60.0	55.5	0.5
	Negative	70	0						
Rapid response COVID-19 IgG/IgM test cassette (whole blood/serum/plasma)	Positive	70	0	43	69.4-88.2	100	70.0-90.0	72.5	0.4
	Negative	20	44						
COVID-19 IgM-IgG combined antibody rapid test	Positive	40	44	44	30.6-58.3	0	60.0-80.2	67.4	0.5
	Negative	50	0						
iChroma COVID-19 Ab	Positive	14	40	15	10.6-18.2	0	30.1-40.0	35.5	0.1
	Negative	80	0						

The Kappa coefficient is a measure of inter-rater reliability or agreement that is used to assess gold standard (RT-PCR) and determine agreement between five antibody detecting assay; n (134) x positive samples by RT-PCR of health care workers and n (134) negative samples by RT-PCR which serves as control

## Discussion

Evidence shows that there are enormous challenges in diagnostic approaches that require rapid and accurate identification of cases of SARS-CoV-2 infections and asymptomatic cases, however, enormous constraints in diagnostics that can rapidly and accurately identify infected persons exist in low resource settings. Approaches that can detect disease progression in order to classify patients for appropriate care and that can thereby prevent exacerbation of the disease, have been recommended [8].

Diagnostic testing is a major component of outbreak detection and emergency response. At the beginning of the coronavirus outbreak globally, there was pressure to expand to an effective response to the testing capacity for the novel coronavirus [8]. In some countries there was a need to get clearance before test kits could be shipped in deployed and approved for use [9]. As the public health institute coordinating the coronavirus pandemic response in Nigeria, the Nigeria Centre for Disease Control (NCDC) set up and scaled up diagnostic capacity for testing across the country.

As part of the scaling up it was important to use best practices for timely detection of the virus causing the pandemic. The gold standard for SARS-CoV-2 detection is RT-PCR, however, to scale up testing capacity with a good TAT, it was necessary to incorporate use of rapid test kits which could be adopted to increase coverage. Based on the results of this study, the 2019-nCoV IgG/IgM antibody determination kit and the rapid response COVID-19 IgG/IgM test cassette had low sensitivity, optimal sensitivity and above average accuracy compared to RT-PCR. Though the COVID-19 IgM-IgG combined antibody rapid test had fair sensitivity, above average accuracy and low sensitivity alongside the other two kits with low specificity. These screening test kits´ sensitivity were much lower than recently conducted validation studies in Sweden and China [10]. Though the optimal sensitivity specificity was the same for the study in Sweden and higher than the studies in China, the test kits however, this study did not differentiate for IgG or IgM sensitivity separately, which was the case in the European and Chinese [10].

In another study in China where a combined IgG and IgM test kit was assessed and validated, the test´ sensitivity was more than two times higher than the sensitivity values in this study, accuracy was lower in this study compared to the previous study in China specificity was however higher in this study [10]. This study validated five combined IgG/IgM antibody-based detection tests and compared them to RT-PCR. With the overwhelming number of SARS-CoV-2 cases and potentially large numbers of asymptomatic cases, the use of RDT Kits with high sensitivity, specificity and accuracy is essential to detecting and tackling SARS-CoV-2 infections.

## Conclusion

The huge impact of the SARS-CoV-2 emergence in public health justifies extensive sero-epidemiological studies to survey its spread in various populations and numerous settings. There is a burst of serologic assays rolling out in different formats, including simple rapid tests. Our study shows that specificity may be highly variable among available immunoassays for antibody to SARS-CoV-2. Poor specificity of an assay in a population where prevalence and incidence of SARS-CoV-2 are low will lead to irrelevant data. Our study, as others, stresses on the absolute necessity to use only carefully validated assays to provide epidemiological data useful to public health decision makers.

**Limitations:** the data analyzed in this study is for a sub-population and may influence the generalizability of this results, compared in a larger population.

### What is known about this topic

- False negative results are possible in pauci-symptomatic subjects is common using rapid SARS-COV-2 kits;
- Several commercial assays detecting similar class of SARS-CoV-2 -specific antibodies.

### What this study adds

- Careful validation of the assay panel is required to provide epidemiological data useful to public health decision makers;
- Discrepancies between assays occurring mainly in this patient category, they should be the target of future studies aimed at correlating the data with the kinetics of N and S-specific antibodies, as well as their neutralizing capacity.
